# Identification of Immune-Linked Hub Genes and Diagnostic Model Construction in Schizophrenia

**DOI:** 10.1007/s12031-023-02138-7

**Published:** 2023-08-08

**Authors:** Kun Lian, Zonglin Shen, Runxu Yang, Jing Ye, Binli Shang, Lei Dong, Hongfang Li, Jiabing Wu, Yuqi Cheng, Xiufeng Xu

**Affiliations:** 1 Department of Psychiatry, First Affiliated Hospital of Kunming Medical University, Kunming, 650000 Yunnan China; 2 Sleep Medical Center, The First People’s Hospital of Yunnan, Kunming, 650101 Yunnan China; 3 Lincang Psychiatric Hospital, Lincang, 677000 Yunnan China; 4 Yunnan Clinical Research Center for Mental Disorders, Kunming, 650000 Yunnan China

**Keywords:** Schizophrenia, WGCNA, LASSO, Immune cell, Hub genes

## Abstract

Schizophrenia (SCZ) is a prevalent, severe, and persistent mental disorder with an unknown etiology. Growing evidence indicates that immunological dysfunction is vital in the development of SCZ. Our study aims to uncover potential immune-linked hub genes and immune infiltration characteristics of SCZ, as well as to develop a diagnostic model based on immune-linked central genes. GSE38484 and GSE54913 chip expression data for patients with SCZ and healthy controls were retrieved. Weighted gene co-expression network analysis (WGCNA) was performed to identify major module genes and critical immune-linked genes. Functional enrichment analysis was conducted to elucidate the involvement of key genes in the immunological response to SCZ, along with the examination of their protein interactions. Moreover, 202 peripheral blood samples were examined using the single-sample gene set enrichment analysis (ssGSEA) method to detect distinct immune cell types. Hub immune-linked genes in SCZ were identified using the minimal absolute contraction and selection operator analysis. Receptor profiles of central immune-linked genes were analyzed to distinguish the two groups. Finally, the association between immune-linked hub genes and various types of immune cells was assessed. Our findings revealed ten immune cell types and nine key genes involved in SCZ, including effector memory CD4+ T cells, activated CD8+ T cells, mast cells, naive CD8+ T cells, PBMC, type 17 helper cells (Th17), central memory CD8+ T cells, CD56 bright NK cells, memory B cells, and regulatory T cells. Diagnostic models constructed using LASSO regression exhibited an average area under the curve (AUC) of 0.866. Our results indicate immunological dysfunction as a factor in the development of SCZ. *ASGR2*, *ADRM1*, *AHANK*, *S100A8*, *FUCA1*, *AKNA*, *GATA3*, *AHCYL2*, and *PTRH2* are the key regulatory genes of immune cells, highlighting their potential as novel therapeutic targets for SCZ.

**Supplementary Information:**

The online version contains supplementary material available at 10.1007/s12031-023-02138-7.

## Introduction

Schizophrenia (SCZ) is a complex mental condition strongly influenced by inheritance^.^202 peripheral blood sample (Marshall et al. 2017; Cheng et al. 2020). It is characterized by persistent periods of psychotic disorientation, behavioral abnormalities, and cognitive impairment (Owen et al. 2016; Gaebel and Zielasek 2015). The global frequency of SCZ is close to 1%, with a higher incidence among young adults (Wambua et al. 2020; Pristed et al. 2017). The pathophysiology of SCZ is multifaceted and relies on the neurodevelopmental, dopamine, and glutamate hypotheses, although these hypotheses do not fully explain all aspects of the disorder (Guan et al. 2022; Bellani et al. 2020). Medical studies indicate that immunological imbalance may also be critical in the development of SCZ, as indicated by elevated plasma concentrations of CRP and inflammatory markers such as IL-6 and TNF-α in persons with acute SCZ (Zamanpoor et al. 2020; Malavia et al. 2017).

Immune system dysfunction, including inflammatory processes and abnormal immune cell blood parameters, has been observed in individuals with SCZ (Michel et al. 2012; Stogios 2021). Multiple studies have demonstrated the associations between immune system abnormalities and the pathophysiology of SCZ (Ilavská et al. 2012). Increased levels of pro-inflammatory markers and cytokines were observed in peripheral blood, cerebrospinal fluid, and tissue samples of patients with SCZ, indicating the activation of adaptive immune responses and involvement of brain immunity (Wang and Miller 2018; Goldsmith et al. 2016). Kim et al. created two immune/inflammation-related co-expression modules using RNA-seq data of SCZ from the Stanley Neuropathology Consortium Comprehensive Database (SNCID) that was correlated with disease states (Kim et al. 2016). Sanders et al. conducted RNA-seq analysis and meta-analysis of combined chip data through lymphoblast cell lines of European pedigree samples from patients with SCZ and CTL populations and found 1058 differentially expressed genes in response to various emotional states, with significant enrichment in immune-linked pathways (Sanders et al. 2017). These studies indicate the involvement of multiple immune-related signaling pathways in patients with SCZ that reflect immunological function and inflammation, such as interleukin and natural killer cell signaling, NF-κB signaling, and B cell signaling, as revealed by a PPI network analysis (Malavia et al. 2017). Genetic evidence also supports the involvement of immune gene variation in SCZ, further substantiating the immunological theory (Zamanpoor 2020). Additionally, adjuvant treatment with nonsteroidal anti-inflammatory drugs (NSAIDs) can dramatically ease psychopathological symptoms in patients with SCZ, indicating a potential role of inflammation in the pathogenesis of SCZ (Birnbaum and Weinberger 2020). Therefore, further investigation into the role of immune cell-regulating genes in SCZ is warranted.

In this study, we employed bioinformatics techniques to identify potential immune-linked hub genes and immune infiltration modes of SCZ. We performed WGCNA to identify module genes and obtained a collection of immune-linked genes specific to 28 different types of peripheral immune cells (Supplementary Table 1) (Charoentong et al. 2017). Functional enrichment and protein interaction network analyses were performed on hub immune-linked genes obtained from the intersection of immune-linked genes and major module genes. Furthermore, we investigated the immune infiltration patterns using the single-sample gene set enrichment analysis (ssGSEA) algorithm and employed the receiver operating characteristic (ROC) curve analysis of immune-associated hub genes to discriminate patients with SCZ from controls. Our study also analyzed the interplay between immune-linked hub genes and distinct immune cell types. We further validated the expression of genes involved in immunological hub function using the GSE54913 dataset. The identification of key immune-related genes and immune infiltration characteristics in SCZ may provide valuable insights into potential therapeutic targets for the disorder.

## Materials and Methods

### Data Processing

**Fig. 1:**
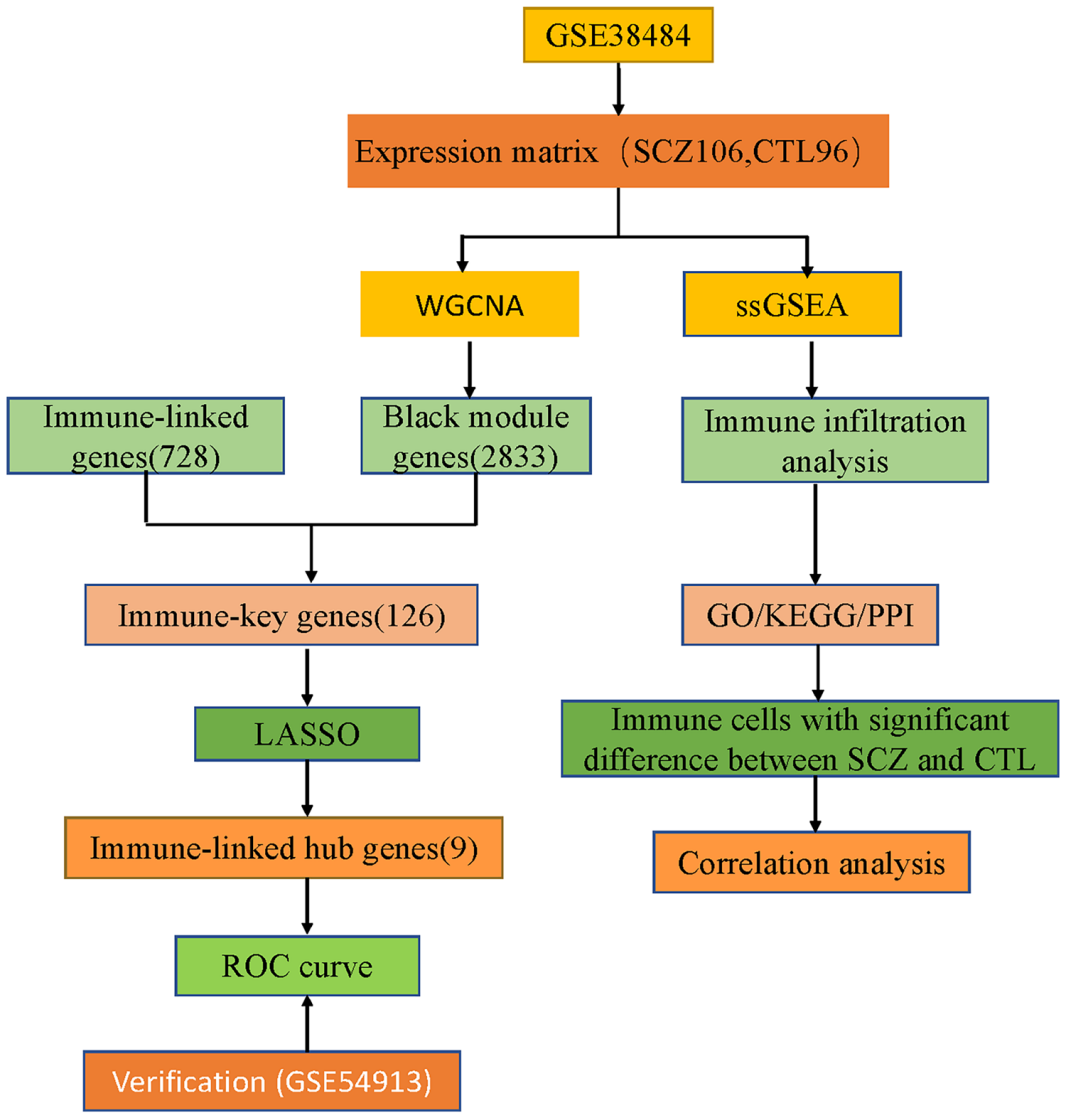
Row chart of the study

The row chart of the study is described in Fig. 1.

The study utilized two datasets, GSE38484 and GSE54913, retrieved from the NCBI website (www.ncbi.nlm.nih.gov). A total of 202 samples were extracted from GSE38484, with 106 samples from patients with SCZ and 96 samples from healthy individuals (regarded as healthy controls). To mitigate the influence of batch effects in the expression data, batch processing was performed using the ComBat function, available in the “sva” package of R software version 4.1.0 (https://www.bioconductor.org/).

### WGCNA Analysis

The WGCNA analysis was performed using an online tool (http://sangerbox.com). All 20,188 genes were included in the analysis using the “WGCNA” program. First, outlier data points were removed, and hierarchical clustering was conducted. Subsequently, a similarity matrix and a scale-free topology were created. The dynamic tree-cutting method was used to create co-expression modules, each containing a minimum of 30 genes. Calculations were made to determine the ME values for each module. Furthermore, the correlation coefficient, as well as the *P*-value between the ME value and the clinical trait phenotype, was determined. A *P*-value < 0.05 was considered significant for SCZ modules. WGCNA constructs scale-free co-expression networks with a sensitivity of 3 and incorporates low-sensitivity modules into these networks.

### Immune Infiltration Analysis

The gene expression profiles of each sample were converted into an immune gene subset enrichment profile using ssGSEA to evaluate the infiltration of 26 immune cells. The immune cell infiltration in the substantia nigra was calculated using the “GSEA” R package. Subsequently, the SCZ and control samples were fitted into the immune infiltration analysis using the ssGSEA method. Statistical analysis was performed to identify significant differences in immune cell infiltration between the two groups, with a significance threshold set at *P* < 0.05.

### Immune Key Gene Identification and Functional Enrichment Analysis

A total of 782 immune-linked genes derived from 26 distinct types of central immune cells were investigated. To identify the key immune-related genes, the WGCNA-screened immune-related genes were intersected with the key module genes. Functional enrichment analysis was performed to assess the putative biological activities of the identified immune genes. The R programs “cluster map” and “GOplot” were employed to perform GO enrichment and KEGG pathway analyses. The study of 782 genes associated with the immune system, taken from 26 distinct types of cells found in the peripheral immune system. The immune-related genes that passed the WGCNA screening were intersected with the key module genes to obtain the hub immune-related genes. Graphic representations of the GO and KEGG pathways were included in the study. Protein–protein interactions were performed using the online resource available at https://cn.string-db.org/. Finally, Cytoscape was used to visualize and interpret the PPI findings.

### Immune-Linked Hub Genes Identified Using LASSO Logistic Regression

To identify new immune-linked hub genes in SCZ, LASSO logistic regression was employed, which offers high predictive value but low correlation for high-dimensional data. The expression levels of critical immune genes and clinical characteristics were analyzed using LASSO logistic regression. Wind and rain have carried the nine genes displayed in the image that were obtained using Omics hare Tools (https://www.omicshare.com/tools/) (Shen et al. 2022). Furthermore, the ROC curve assessment of immune-associated hub genes was employed to distinguish patients with SCZ from healthy controls using the “pROC” R package. Finally, the immune system regulation by various immune cells linked to the hub genes was investigated.

### Validation of Hub Genes

Differential expression of immune-linked hub genes between SCZ and controls was validated using the GSE54913 dataset. The differences in hub gene expression were visualized using boxplots generated with the “tiny array” package’s boxplot function. The Kruskal–Wallis test was performed to compare the expression levels of hub genes between the SCZ group and the control group. A *P*-value less than 0.05 was regarded as statistically significant. Finally, the diagnostic model was confirmed using logistic regression analysis, considering the median expression levels of nine immune-linked hub genes.

## Results

### Modules for Gene Co-Expression Identification

**Fig. 2:**
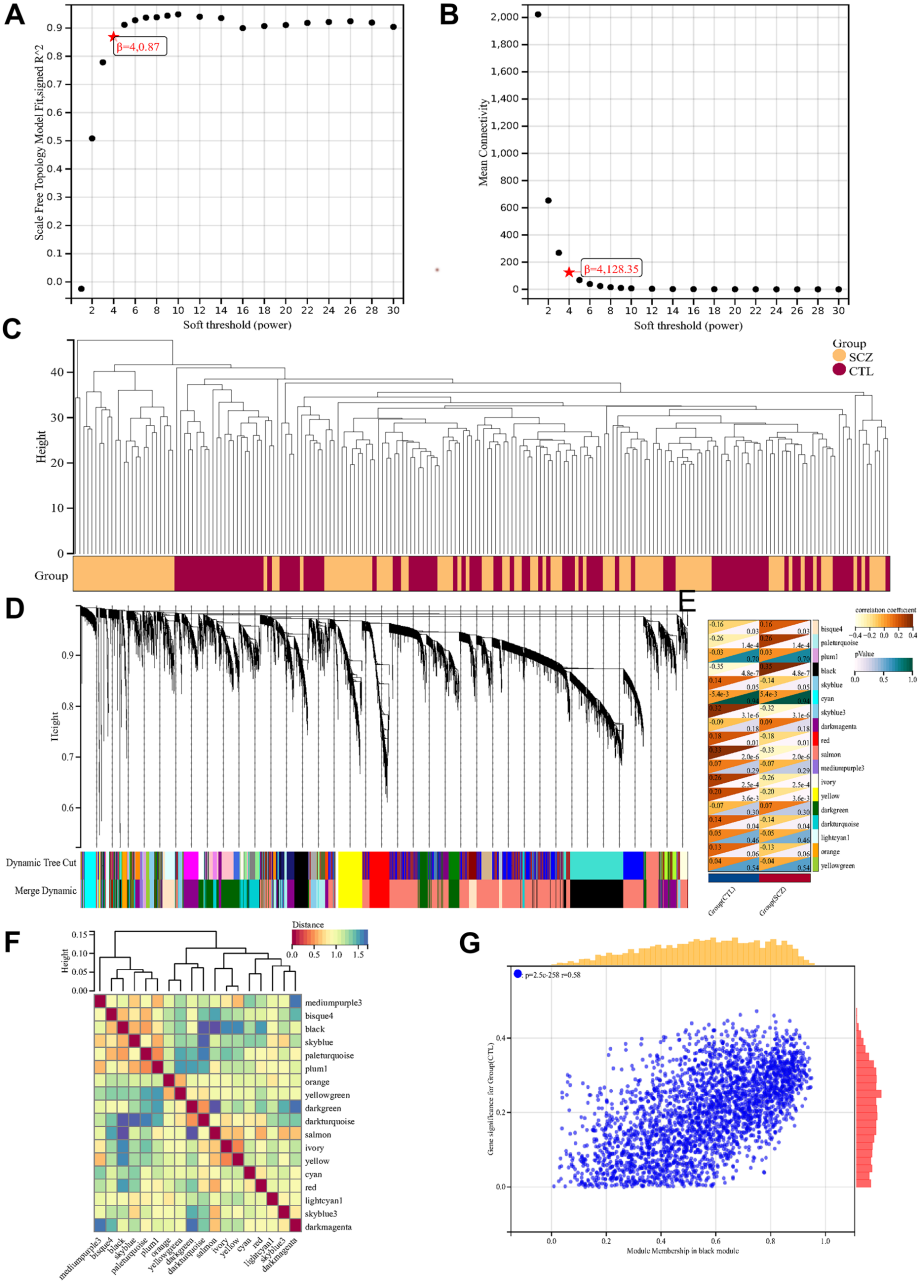
Results of the WGCNA. **A** The corresponding scale-free topological model fit indices at different soft threshold powers. **B** The corresponding mean connectivity values at different soft threshold powers. **C** The heat maps of clinical features. **D** Cluster dendrogram of genes. **E**) Correlations between different modules and clinical traits. **F** Vector clustering of module feature. **G** Correlation of module membership and gene significance in the brown module

After accounting for batch effects, we identified 20,188 genes using the empirical Bayesian technique ComBat (Supplementary Table 2). The co-expression network was built using the 2833 most variable genes from a dataset of 202 samples. At a sensitivity of 4, the average connection value was 128.35, and the independence was 0.87 (Fig. 2A and B). We first used stratified clustering for each sample and generated heat maps illustrating the clinical features (Fig. 2C). Modules with fewer than 25% of their genes and fewer than 30 common genes were combined to form larger modules. Finally, 18 co-expression modules were identified, while the gray module was shown to be incorrect (Fig. 2D). A heat map of the related gene network and clinical features was created based on the ME values of the modules (Fig. 2E). Subsequently, we analyzed the association between each sample and the identified module using a heat map (Fig. 2F). The strongest positive correlation was found between the black modules and SCZ (*r* = 0.58, *P* = 2.5e258) (Fig. 2G); therefore, we chose them as the primary modules to examine. Subsequently, 121 black module hub genes correlated with the MM, GS, and weight threshold (Supplementary Table 3).

### Immune Infiltration Analysis in SCZ

**Fig. 3:**
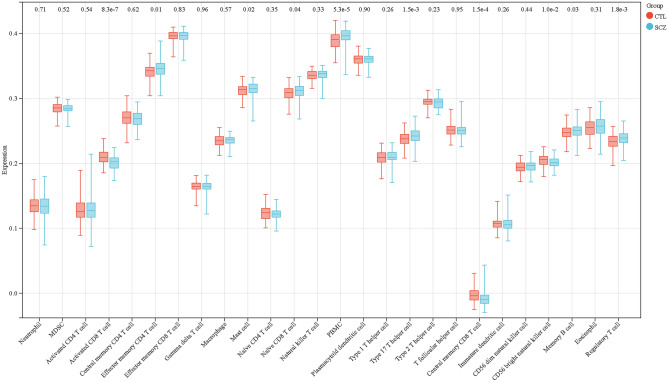
The 26 immune cell infiltration analysis of substantia nigra between SCZ patients and healthy controls

To investigate immune infiltration in SCZ, we employed the ssGSEA method to evaluate the presence of 26 distinct immune cell types within the substantia nigra of SCZ and control samples. We found significantly higher levels of activated CD8+ T cells, effector memory CD4+ T cells, mast cells, naïve CD8+ T cells, peripheral blood mononuclear cells (PBMC), Th17, central memory CD8+ T cells, CD56 bright NK cells, memory B cells, and regulatory T cells in the SCZ group compared to the control group (Fig. 3). Conversely, immature dendritic cell (dc) infiltration was lower in patients with SCZ across various immune cell types. Our immune infiltration analysis revealed 10 distinct infiltrating immune cell types between SCZ and control samples. These cell types include activated CD8+ T cells, effector memory CD4+ T cells, mast cells, naive CD8+ T cells, peripheral blood mononuclear cells (PBMC), type 17 helper cells (Th17), central memory CD8+ T cells, CD56 bright NK cells, memory B cells, and regulatory T cells. Finally, nine hub genes were eliminated using LASSO logistic regression: *ASGR2*, *ADRM1*, *AHANK*, *S100A8*, *FUCA1*, *AKNA*, *GATA3*, *AHCYL2*, and *PTRH2*.

### Identification of Immune Key Genes, Functional Enrichment, and PPI Analysis

**Fig. 4:**
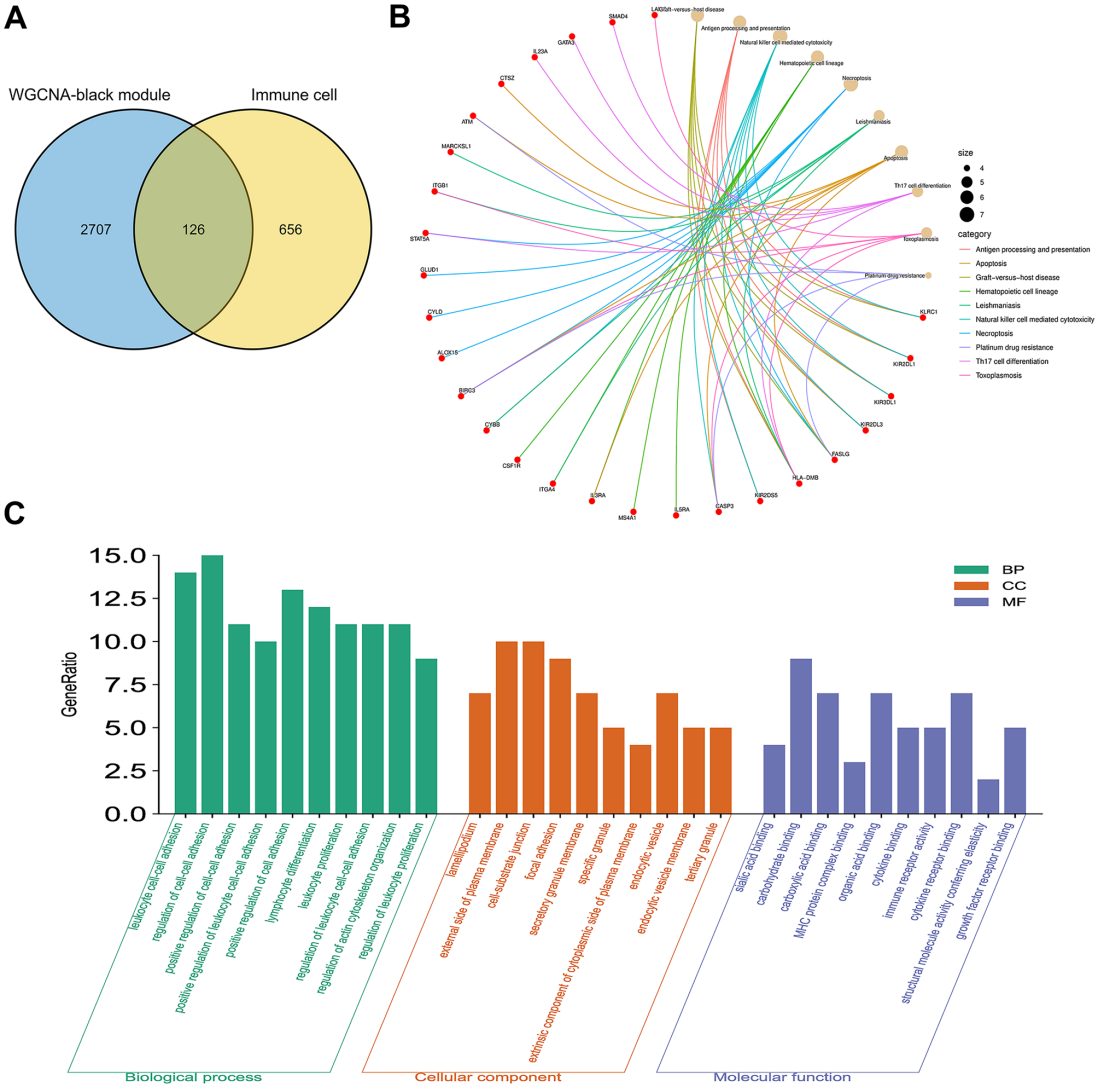
Hub genes and GO/KEGG analysis. **A** Venn diagram of genes screened via WGCNA and immune-related genes dataset. **B** KEGG pathway enrichment analysis enriched by the hub genes. **C** GO: BP, CC, MF in which the hub genes were involved

**Fig. 5:**
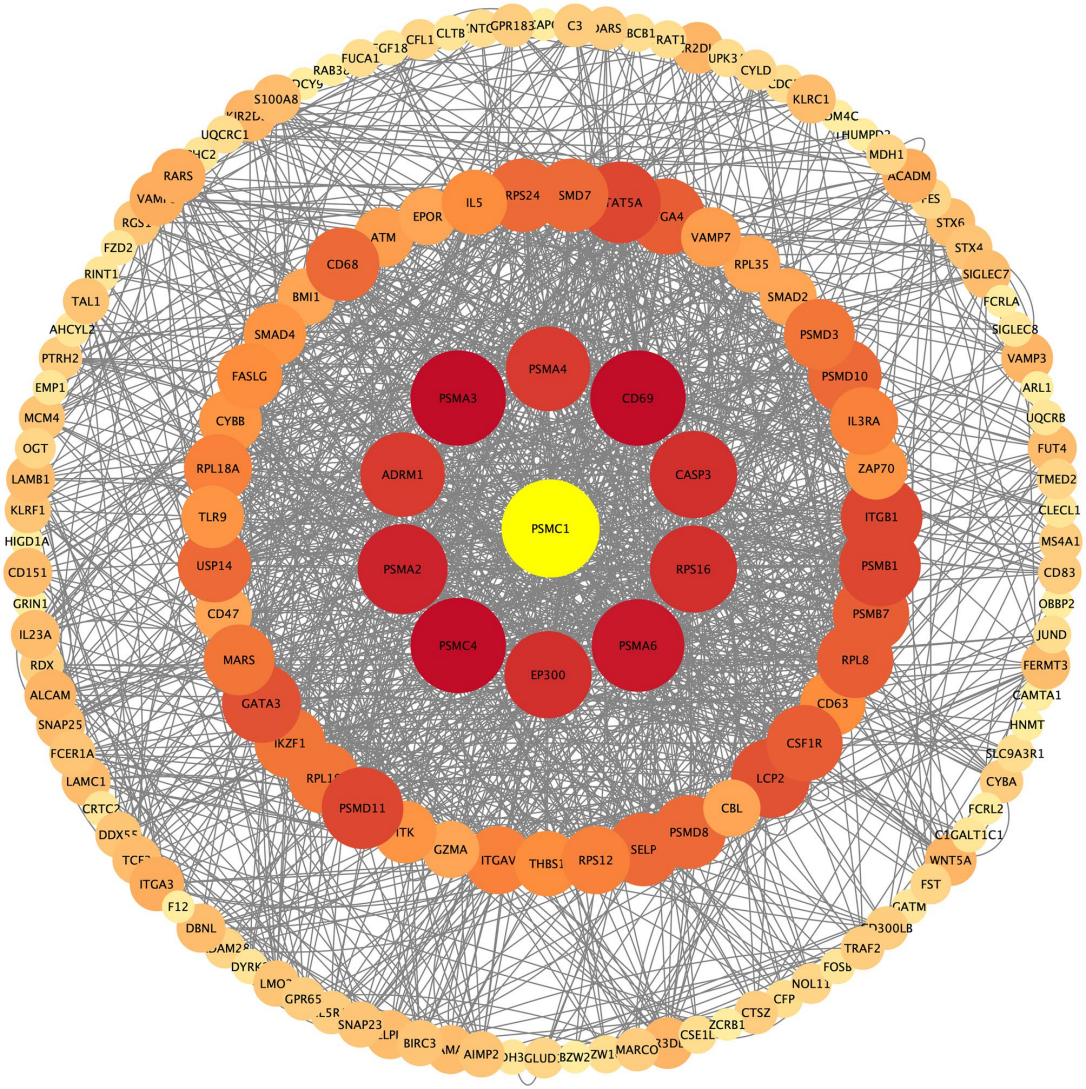
PPI of 126 immune-linked key genes in SCZ

**Fig. 6:**
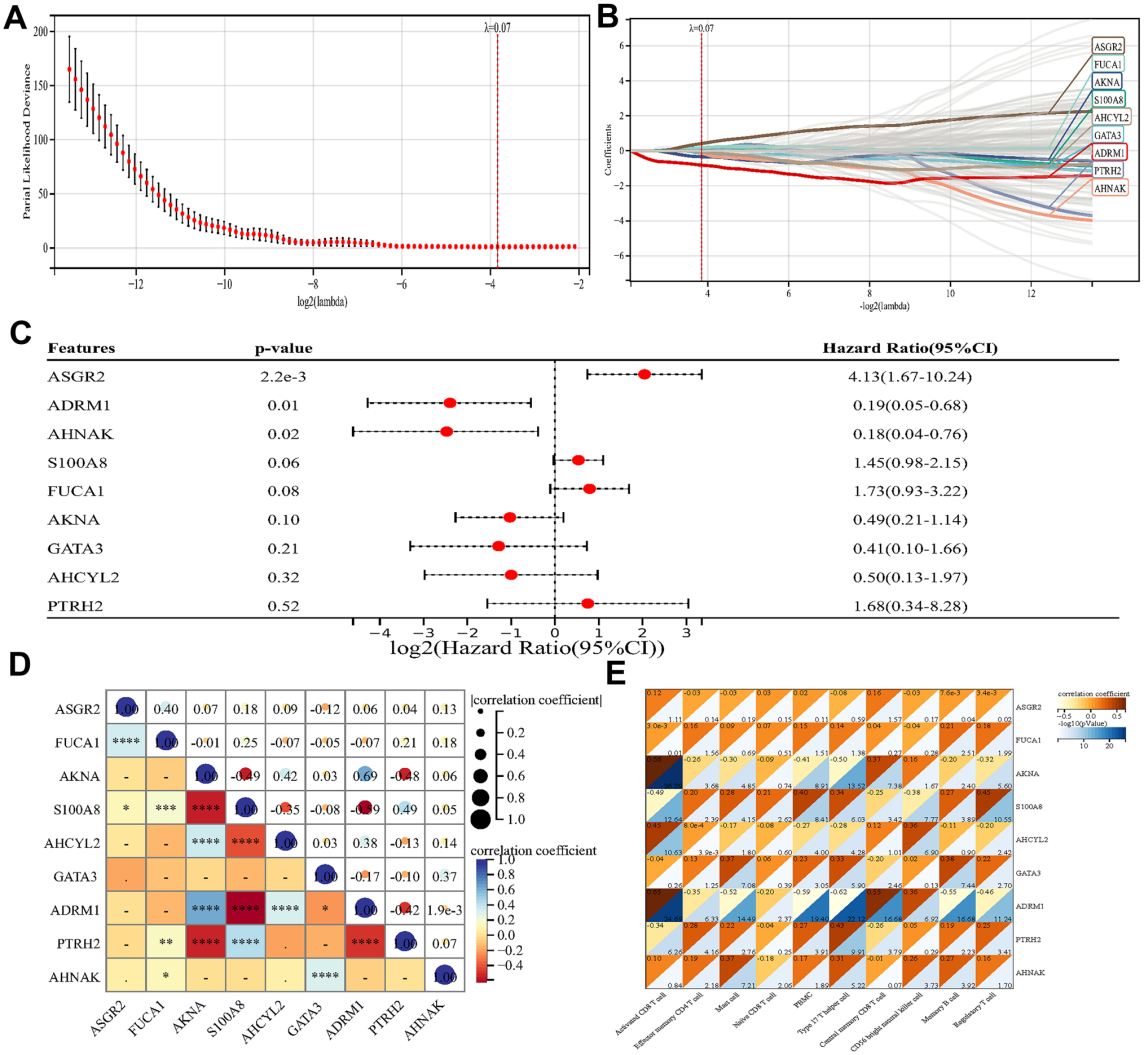
Identification of immune-related hub genes via the LASSO model. **A** Tuning parameter (lambda) selection in the LASSO regression model. **B** The LASSO coefficient profiles. **C** Cox analysis of nine hub genes in 202 samples. **D** The relationship among immune-related hub genes. **E** The relationship between immune-related hub genes and immune cells. *p < 0.05 and **p < 0.01

Our study included immune-linked genes derived from 26 distinct peripheral immune cell types, yielding 782 immune-related genes (Supplementary Dataset 1). By intersecting these immune-linked genes with the black module genes, we identified 126 key immune genes depicted (Fig. 4A). To gain insights into the biological functions and pathways associated with these key immune genes, we performed GO and KEGG pathway enrichment analyses. The GO enrichment analysis revealed enrichment in immune-linked biological processes, such as the control of leukocyte–cell adhesion, lymphocyte proliferation, actin cytoskeleton organization, and immune receptor binding (Fig. 5C). Furthermore, the KEGG pathway enrichment analysis demonstrated that these genes are primarily involved in immunological pathways, such as cell adhesion molecules, NK-mediated cytotoxicity, Th17 cell development, necrotizing apoptosis, and apoptosis (Fig. 5B). Finally, we used PPI network analysis from the online analysis and imported them into Cytoscape to establish networks between these proteins (Fig. 6).

### Immune-Linked Hub Gene Screening and Immune Cell Correlation Analysis

To identify central genes associated with the immune system in SCZ, we built a LASSO model using immune-critical gene expression levels and clinical features (SCZ vs. control) across the entire dataset (Fig. 6A). The regression coefficient for nine immune-related hub genes, namely, *ASGR2, ADRM1, AHANK, S100A8, FUCA1, AKNA, GATA3, AHCYL2*, and *PTRH2*, was nonzero, as assessed by the minimal value of lambda (Fig. 6B). Moreover, Cox analysis was utilized to establish the predictive value of these nine genes in 202 samples (Fig. 6C) (Supplementary Table 4). In the network of immune-linked hub genes, ASGR2 was correlated positively with FUCA1 and negatively with GATA3. Additionally, *SA100A8*, *PTRH2*, and *AHANK* exhibited negative associations with *FUCA1*. *AHCYL2* and *ADRM1* were favorably connected with *AKNA*, while *S100A8* and *PTRH2* were adversely correlated. *S100A8* had a positive association with *PTRH2* but a negative association with *AHCYL2* and *ADRM1*. A positive correlation was observed between *AHCYL2* and *ADRM1*. *GATA3* was associated positively with *AHANK* and negatively with *ADRM1*. Furthermore, *ADRM1* displayed an inverse relationship with *PTRH2* (Fig. 6D). Finally, ten immune cell types and nine major immune-related genes were analyzed for correlations (Fig. 6E).

### Expression of Immune-Linked Hub Gene

**Fig. 7:**
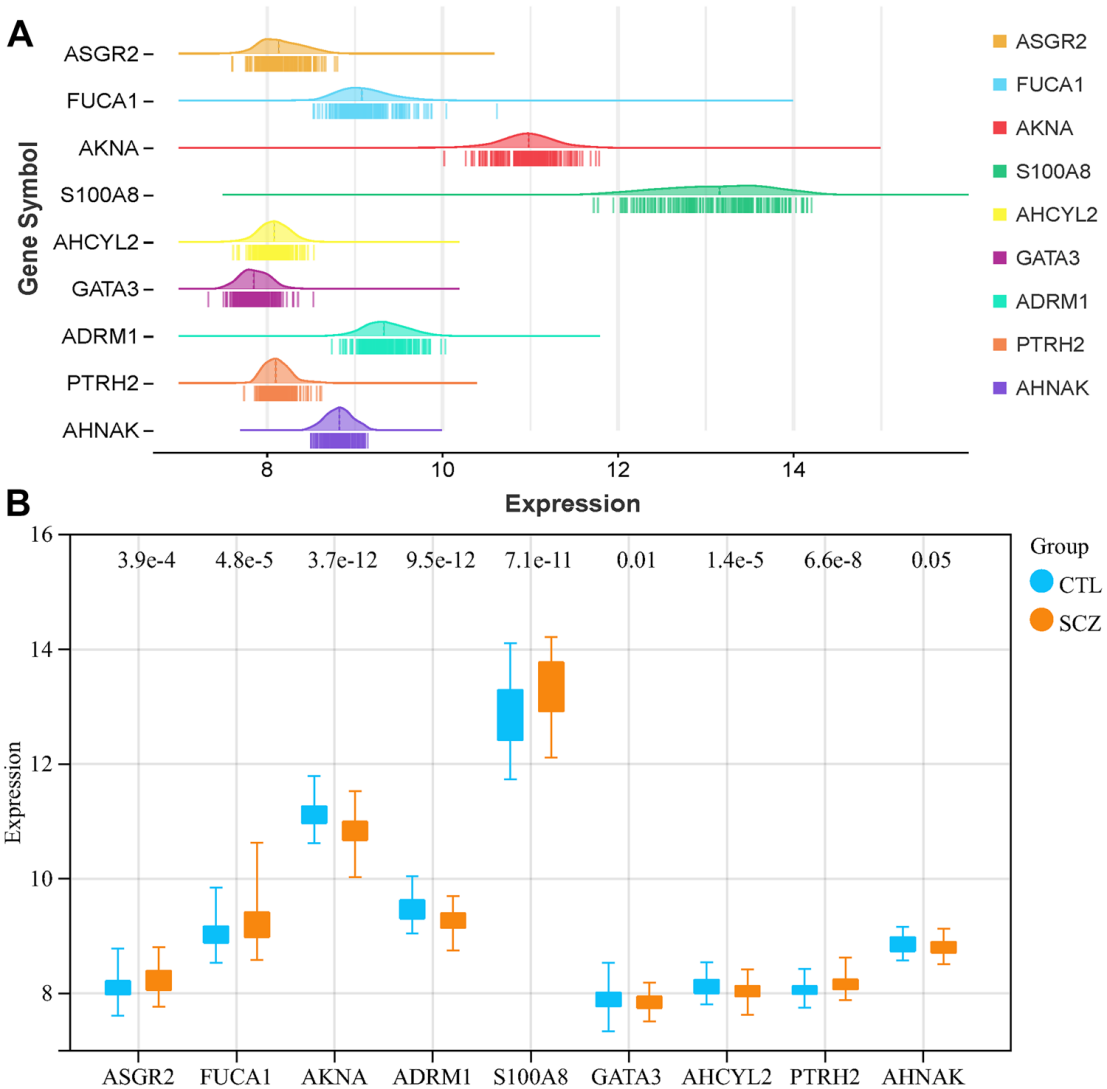
**A** The expression level of immune-linked hub genes in 202 samples. **B** The difference in expression of the SCZ and CTL

To determine the expression levels of central immune-linked genes, we analyzed 202 samples, including 96 healthy controls and 106 patients with SCZ. The deformation cloud and rain diagram were used to visualize the expression patterns, combining the half violin diagram (the core density curve), boxplot, and jitter scatter diagram (Fig. 7A). The expression levels of *AKNA*, *ADRM1*, *GTAT3*, *AHCYL2*, and *AHANK* were also significantly downregulated in the SCZ group compared to the controls, while the expression levels of *ASGR2*, *FUCA1*, *S100A8*, and *PTRH2* exhibited significant upregulation (Fig. 7B).

### ROC Curve of Immune-Linked Hub Genes

**Fig. 8:**
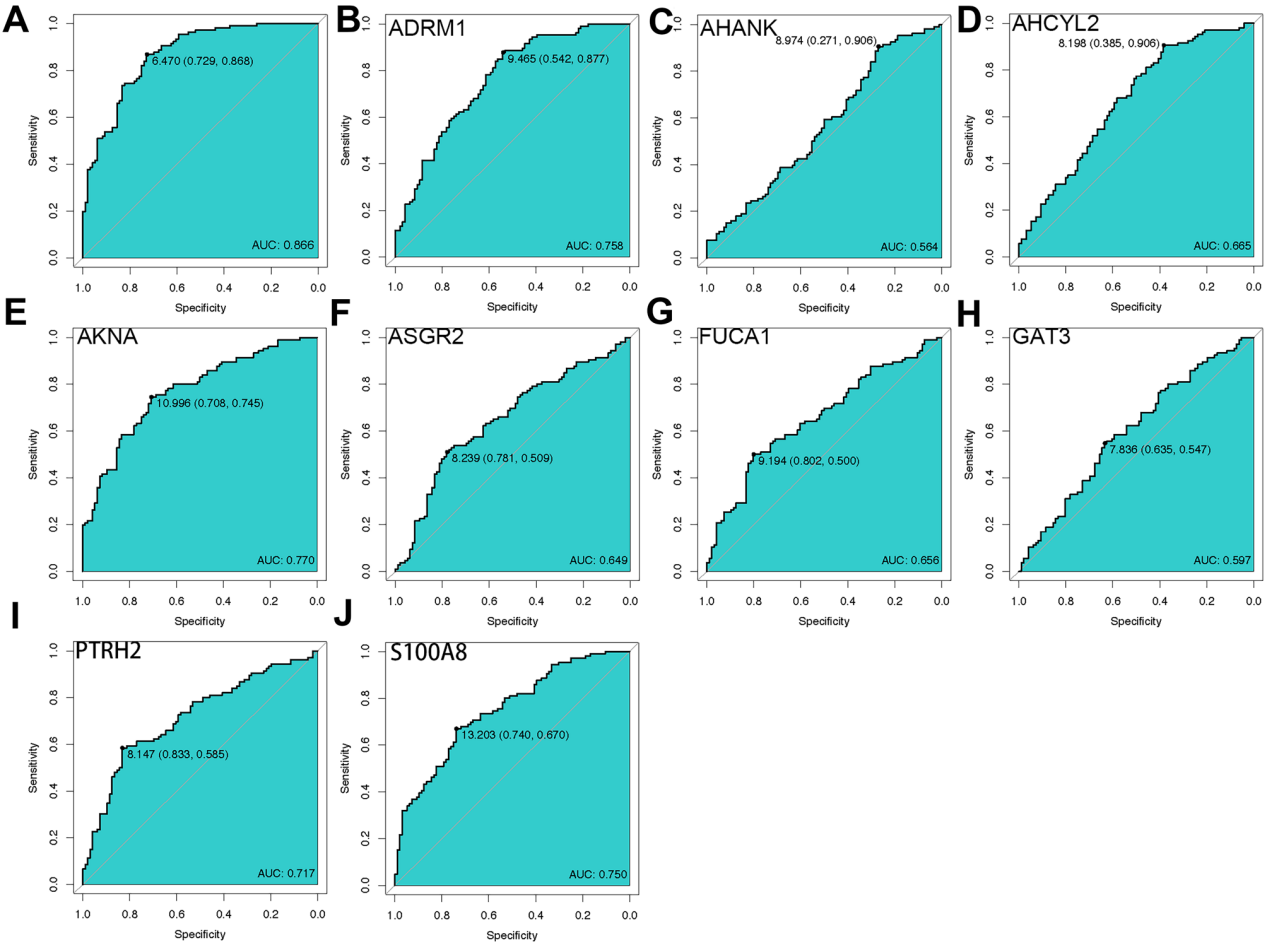
ROC curve of immune-related hub genes. **A** The ROC curve of nine immune-linked hub genes. **B**–**J** The ROC curve of each immune-related hub gene

We conducted an ROC curve analysis to assess the ability of immune-linked core genes to distinguish patients with SCZ from healthy controls. The area under the curve (AUC) for the model containing all nine genes was 0.866 (Fig. 8A). Furthermore, the AUC values for individual immune-linked hub genes (*ADRM1*, *AHANK*, *AHCYL2*, *AKNA*, *ASGR2*, *FUCA1*, *GAT3*, *PTRH2*, and *S100A8*) were 0.758, 0.564, 0.665, 0.770, 0.649, 0.656, 0.597, 0.717, and 0.750, respectively (Fig. 8B–J).

### GSE54913 for Immune Hub Gene Validation

**Fig. 9:**
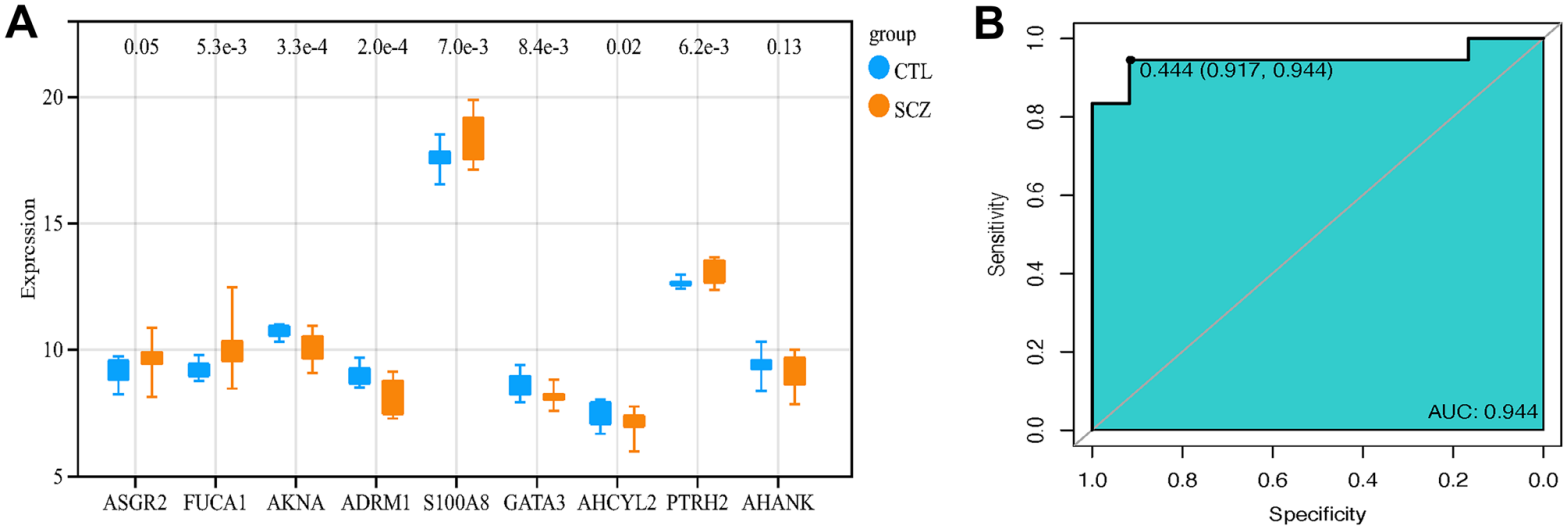
**A** The difference in expression of the SCZ and CTL in GSE53987. **B** The ROC curve of nine immune-linked hub genes in GSE53987

To validate the expression levels of immune-linked core genes, we examined the GSE54913 dataset, which included 18 patients with SCZ and 12 healthy controls. We found that *ASGR2*, *FUCA1*, *S100A8*, and *PTRH2* were significantly upregulated in patients with SCZ, while *AKNA*, *ADRM1*, *GATA3*, and *AHCYL2* were significantly downregulated (Fig. 9A). On the other hand, *AHANK* expression did not show significant differences. Additionally, we also performed ROC curve analysis on this dataset for each of these nine genes based on their average expression levels (Fig. 9B).

## Discussion

Schizophrenia is a brain disorder characterized by impaired cognitive and behavioral processes (Maj et al. 2021; Su et al. 2015). The neuro-immune theory suggests that immune system components, such as the complement cascade, are involved in SCZ based on genetic investigations (Das et al. 2018). Moreover, patients with SCZ often exhibit elevated levels of pro-inflammatory cytokines, indicating abnormalities in the inflammatory and immune systems that may contribute to the pathophysiology of the disorder (Maes et al. 2021; Vasupanrajit et al. 2021). Current SCZ biomarkers primarily include (1) risk assessment markers, such as genetic markers; (2) clinical diagnosis and disease course monitoring markers, such as inflammatory markers; and (3) efficacy evaluation markers, such as pharmacogenomic markers (Lin et al. 2022). A mouse model of MAM (mitotoxin methylazoxymethanol acetate) has been developed to reproduce epigenetic and transcriptional abnormalities associated with SCZ in the hippocampus and prefrontal cortex (Du et al. 2019a). Further investigation revealed significant enrichment in the pathways involved in the synthesis of the amino acid phenylalanine, tyrosine, and tryptophan, as well as glycerophospholipid metabolism among the 25 differentially expressed metabolites in SCZ (Du et al. 2020). Additionally, patients with SCZ exhibited differential expression profiles of blood exosome-derived miRNAs. Genes involved in protein glycosylation, neurodevelopment, neurotransmission, and synaptic plasticity were enriched in a bioinformatics analysis of SCZ-affected miRNAs and miRNA co-expression module targets; these genes included*BDNF*, *GALNT15*, *CDC42*, and *DISC1* (Du et al. 2019b) One study analyzed gene expression data from postmortem brain samples of patients with SCZ and developed a risk prediction model for early diagnosis of SCZ using machine learning methods (Li et al. 2022). In this study, we used bioinformatics to identify potential biological targets for diagnosing and treating SCZ by detecting immune-related central genes and immune infiltration patterns in the peripheral blood of patients with SCZ.

Analysis of the black module, consisting of 2,833 genes, revealed an overlap between the genes that make up the black module and the 782 genes involved in the immune system, 126 of which are considered immune-key genes. The GO/KEGG enrichment analysis revealed that the immunological pathway was enriched in the critical genes for immune-mediated cytotoxicity, Th17 differentiation, NK-mediated cytotoxicity, and actin cytoskeleton organization. The PSMC gene family exhibited the most extensive protein interaction based on PPI analysis.

Among the immune-linked hub genes, the *ASGR2* gene at chromosome 17p13.1 encodes a transmembrane protein. *ASGR2* and *ASGR1*combine to produce heteromorphic or homomorphic polymers. The primary function of ASGR2 is to identify and endocytose terminal glycoproteins carrying acetylgalactose or galactose and to eliminate toxic glycocholate created by normal tissue metabolism, damage, pathological process, and other causes (Clinical Phenotype and Pathogenic Mechanisms 2022). On the other hand,*FUCA1* is a glycol-based hydrolase expressed in nerve cells and catabolizes biological macromolecules such as glycoproteins and glycolipids. It has a crucial regulatory role in the immunological response, signal transduction, and embryonic development. The absence of *FUCA1*can result in motor dysfunction, neuronal death, and other neurological conditions (Baudot et al. 2022; Camargo Ortega et al. 2019). Transcriptional regulator*AKNA* is an intracellular protein with an AT-hook mechanism that regulates the transcription of target genes by binding to AT-rich DNA sequences. It regulates immune responses, predominantly expressed in B lymphocytes, T lymphocytes, natural killer cells, and stem cells. Inactivation of the *AKNA*gene can lead to an increase in inflammatory factors, proteases, and chemokines, as well as an inflammatory response mediated by neutrophils (Siddiqa et al. 2001; Kawaguchi et al. 2021). *ADRM1*is a membrane glycoprotein and a polyubiquitin receptor for the 26S proteasome. It could engage in multiple key biochemical processes, such as cell proliferation, differentiation, death, and immunological control through the JAK2-STAT3 signal transduction pathway (Nath et al. 2018; Lu et al. 2020). *S100A8* is a Ca^2+^-binding protein that is typically secreted by activated granulocytes and functions as an inflammatory cytokine by binding to cell surface receptors such as Toll-like receptor 4, CD39, or receptors for end-products of advanced glycation, thereby activating inflammatory signaling and regulating numerous inflammatory disease processes (Willers et al. 2020; Wang et al. 2018). *GATA3*, situated on chromosome 10, is an essential component of Th2 cell development and cytokine production. *GATA3*was confirmed as a master transcription factor associated with Th2 cells, stimulating the synthesis of IL-4, IL-13, and IL-5 in Th2 cells (Wan et al. 2014; Banerjee et al. 2013). *AHCYL2*encodes adenosine homocysteinase-like 2 proteins (SAH2), which affects the alteration of low-density lipoprotein by converting adenosine homocysteine to adenosine and homocysteine (Huang et al. 2022; Parkhitko et al. 2016). *PTRH2*, a 19-kDa protein found on the mitochondria, plasma membrane, endoplasmic reticulum, and Golgi devices of eukaryotic cells modulates Bcl2 expression as well as PI3K/AKT and ERK signaling in response to cell survival, growth, and differentiation. Mutations in *PTRH2*can result in abnormalities of the neurological system, muscles, endocrine system, and others (Corpuz et al. 2020; Hu et al. 2014). The largest protein in the body, AHNAK, is required for calcium signaling during CD4+ T cell activation and plays a role in the cytoskeletal structure, muscle regeneration, and the formation of calcium homeostasis (Jin et al. 2020; Choi et al. 2019).

To further differentiate patients with SCZ from controls, we conducted a ROC curve analysis of core genes involved in the immune system. Our analysis revealed an AUC of 0.886 for the 9-gene model. All immune-related hub genes had AUC values above 0.70; however, the highest values were seen in *ADR1*, *AKNA*, *S100A8*, and *PTRH2*, suggesting their potential as hallmark genes for SCZ.

Furthermore, we explore the correlation between nine core genes and 26 immune cell types. We observed a strong correlation of the hub genes with several immune cells, including activated CD8+ T cells, effector memory CD4+ T cells, mast cells, naive CD8+ T cells, PBMC, Th17, central memory CD8+ T cells, CD56 bright NK cells, memory B cells, and regulatory T cells, indicating their involvement in SCZ pathogenesis.

Collectively, we identified nine immune-linked hub genes closely associated with the pathogenesis of SCZ (*ASGR2*, *ADRM1*, *AHANK*, *S100A8*, *FUCA1*, *AKNA*, *GATA3*, *AHCYL2*, and *PTRH2*) and ten peripheral immune cell types. Hub genes associated with the immune system were mostly associated with biological processes, such as immunological activity, immune cytotoxicity, immune cell adhesion, and immune cytoskeleton. The findings support the growing evidence implicating neural immunity in the pathophysiology of SCZ (Khandaker et al. 2015; Dickerson et al. 2017). Infiltration of immune cells from the periphery and excess activation of microglia can cause inflammation and neuronal injury in the brain. Moreover, studies suggest that SCZ may be caused by an inflammatory response that disrupts the blood–brain barrier and affects neuroinflammation in the central nervous system by controlling the function of microglia and astrocytes (Nakamura et al. 2019; Buckley 2019).

However, the limits of our research must be acknowledged. Firstly, validation of our findings through in vitro experiments is necessary. Secondly, collecting samples from a larger cohort of patients with SCZ is important to investigate immune-related core genes and provide biomarkers for timely medication intervention. Future studies should focus on investigating the involvement of *FUCA1*, *AKNA*, *ADRM1*, *S100A8*, and *GTAT3*. Additionally, efforts should be made to identify and validate biomarkers that could enable the early identification and diagnosis of SCZ, facilitating early intervention strategies and improving patient outcomes.

## Conclusion

The bioinformatics investigation conducted in this study revealed the presence of central immune-related genes in the peripheral blood of patients with SCZ. *ASGR2*, *ADRM1*, *AHANK*, *S100A8*, *FUCA1*, *AKNA*, *GATA3*, *AHCYL2*, and *PTRH2* emerged as potential genes warranting further investigation. These findings contribute to our understanding of the role of immune-linked genes in the pathogenesis of SCZ and provide potential targets for diagnostic and therapeutic interventions in this disorder.

### Supplementary Information

Below is the link to the electronic supplementary material.

Supplementary file1 (XLSX 32 KB)

Supplementary file2 (XLSX 104 KB)

Supplementary file3 (XLSX 23 KB)

Supplementary file4 (TXT 1 KB)

## Data Availability

The data used to support the finding is available in the GEO dataset (GSE38484 and GSE54913).
